# Methods for identifying surgical wound infection after discharge from hospital: a systematic review

**DOI:** 10.1186/1471-2334-6-170

**Published:** 2006-11-27

**Authors:** Emily S Petherick, Jane E Dalton, Peter J Moore, Nicky Cullum

**Affiliations:** 1 Department of Health Sciences, University of York, Seebohm Rowntree Building, York, UK; 2 Centre for Reviews and Dissemination, University of York, York, UK; 3 Department of Surgery, Scunthorpe General Hospital, Scunthorpe, UK

## Abstract

**Background:**

Wound infections are a common complication of surgery that add significantly to the morbidity of patients and costs of treatment. The global trend towards reducing length of hospital stay post-surgery and the increase in day case surgery means that surgical site infections (SSI) will increasingly occur after hospital discharge. Surveillance of SSIs is important because rates of SSI are viewed as a measure of hospital performance, however accurate detection of SSIs post-hospital discharge is not straightforward.

**Methods:**

We conducted a systematic review of methods of post discharge surveillance for surgical wound infection and undertook a national audit of methods of post-discharge surveillance for surgical site infection currently used within United Kingdom NHS Trusts.

**Results:**

Seven reports of six comparative studies which examined the validity of post-discharge surveillance methods were located; these involved different comparisons and some had methodological limitations, making it difficult to identify an optimal method. Several studies evaluated automated screening of electronic records and found this to be a useful strategy for the identification of SSIs that occurred post discharge. The audit identified a wide range of relevant post-discharge surveillance programmes in England, Scotland and Wales and Northern Ireland; however, these programmes used varying approaches for which there is little supporting evidence of validity and/or reliability.

**Conclusion:**

In order to establish robust methods of surveillance for those surgical site infections that occur post discharge, there is a need to develop a method of case ascertainment that is valid and reliable post discharge. Existing research has not identified a valid and reliable method. A standardised definition of wound infection (e.g. that of the Centres for Disease Control) should be used as a basis for developing a feasible, valid and reliable approach to defining post discharge SSI. At a local level, the method used to ascertain post discharge SSI will depend upon the purpose of the surveillance, the nature of available routine data and the resources available.

## Background

Surgical site infections (SSIs) have been estimated to occur in up to 15% of elective surgical patients and approximately 30% of patients whose surgical procedure was classed as contaminated or "dirty" [1]. The proportion of SSIs that are preventable is unknown, however there are wide variations in infection rates and an international drive to minimise them [1]. Accurate, standardised methods of defining and monitoring SSIs are essential in order to identify baseline infection rates, to describe and investigate variations in infection rates, evaluate the effectiveness of interventions, prioritise resource allocation and identify those at highest risk. SSI surveillance is widely employed and often mandatory[2]. Surveillance has been defined as a systematic, ongoing collection, collation and analysis of data and timely dissemination of information to those who need to know so that action can be taken [3]. Surveillance has four discrete components; data collection, data collation, data analysis and dissemination [4]. Regardless of the purpose of surveillance, several key features are required for both the development and ongoing evaluation of a surveillance system, including appropriate case definition, population definition, data sourcing and data collection [5]. Two of the main challenges to ensuring that SSI surveillance adequately incorporates post discharge SSIs are:

• the lack of a validated method of ascertaining cases of SSI post discharge;

• the paucity of studies comparing different methods in a consistent way (large variations in definition of SSIs; staffing, setting, data sources and timings used in existing studies).

A previous systematic review addressed a broad range of questions relating to the measurement and monitoring of adverse events of surgery, including SSIs [1]. This review identified common and potentially avoidable surgical adverse events and examined whether they could be reliably and validly measured. The review concluded that there was:

*"inconsistency in the quality of reporting of postoperative adverse events, limiting comparison of rates over time and between institutions"*.[1]

We sought to build on and update this review in relation to PDS SSI.

Specifically we sought to answer the following three questions:

1. What is the evidence for the validity, reliability and practicality of different methods of case ascertainment and surveillance for SSIs post discharge?

2. What are the features of post discharge surveillance systems used in the UK in terms of coverage; source of denominator data; diagnostic characteristics; other data collected; methods of data collection?

3. What further research is needed to identify a valid, reliable, practical methods of case ascertainment and surveillance for SSIs post discharge?

### Definition of Surgical Site Infection

The most widely used definition of a surgical site infection is probably that described by the CDC [6]. Briefly, a superficial surgical site infection is defined as one that occurs within 30 days of the operative procedure and involves only the skin or subcutaneous tissue of the incision, and at least one of the following:

▪ purulent drainage from the superficial incision;

▪ organisms isolated from an aseptically obtained culture of fluid or tissue from the superficial incision;

▪ at least one of the following signs or symptoms of infection: pain or tenderness, localised swelling, redness or heat and superficial incision is deliberately opened by a surgeon, unless culture of incision is negative;

▪ diagnosis of superficial incisional SSI by the surgeon or attending physician.

## Methods

Different techniques of PDS for SSI were identified and described using a combination of systematic review and a national audit.

### Systematic review

The systematic review sought to answer the question: *What is the current evidence for the validity, reliability and practicality of different systems of PDS of SSI?*

The literature search was based on that undertaken for a previous review (search dates from 1993 to 1999) [1] and was updated to 2004 using the databases shown (Table 1).

**Table 1:** Electronic databases searched for this review

**Database**	**Period/Issue covered**
Medline (OVID)	1999–02/2004
EMBASE	1999–03/2004
CINAHL	1999–03/2004
The Cochrane Library Database of Systematic Reviews	1999–2004 Issue 1

In order to be eligible for inclusion, studies had to:

a) Describe a method of post discharge surveillance for surgical site infection OR

b) compare at least two methods of post discharge surveillance for surgical site infection OR

c) describe an economic evaluation of a post discharge surveillance system for surgical site infection.

Papers were excluded if they met any of the following criteria;

a) Paper gave no indication that post discharge follow up was performed OR

b) post-discharge follow up was carried out for reasons other than for surveillance of surgical wound infection, OR

c) appeared to be routine patient follow up post operation with no methodological detail provided of how post-discharge follow up was achieved.

Two abstractors assessed potentially eligible articles, independently applying the inclusion and exclusion criteria. Any disagreements were solved by discussion. For completeness it was decided to re-abstract and include eligible validation studies from a previous review [1].

### Validation studies

In addition to the inclusion criteria above, in order for validation studies to be eligible for inclusion in this review they had to compare alternative ascertainment techniques, and furthermore patients had to receive both techniques regardless of the results of either method.

The Quality Assessment of Diagnostic Accuracy Studies (QUADAS) tool [7] was used to assess the validity of the studies comparing different methods of case ascertainment and surveillance. The QUADAS tool includes 14 questions about the spectrum of patients studied, selection criteria, test verification, test description, blinding, uninterpretable results, and study withdrawals [7].

### National Audit of current PDS practice in the UK

PDS systems for SSIs currently (May 2004) used in the UK were identified by audit. A brief audit form was sent to infection control personnel within all UK primary care trusts and hospital trusts to identify those Trusts that were undertaking any form of PDS for SSIs.

## Results

### Literature Review

Our literature search overlapped with that of the previous review by one year (1998) (see Table 1) [1]. The search yielded a total of 3,548 article titles and/or abstracts, from which 130 appeared potentially eligible and were ordered for full text assessment.

From the 130 papers assessed in full text, a total of 78 referred to post discharge surveillance for surgical site infections. Of these, 73 papers described a single surveillance programme. No studies were located that looked at the impact on patient outcomes of establishing post discharge wound surveillance. Only three papers reported research that compared different surveillance methods. Three studies located in a previous review [1] and identified as validation studies were re-abstracted for completeness bringing the total number of comparative studies included in this study to six (seven reports).

The methods used to detect post-discharge SSI in the literature were:

• direct observation of the wound by health professional (n = 31)

• telephone interviews with patients (n = 17)

• patient questionnaire (n = 13)

• other methods (n = 21). Other methods used included review of operating logs to examine surgical revisions; cards to be used by patients to notify health care personnel of a surgical site infection; examination of hospital readmission data; review of pharmacy data; and using mixed methods.

• method not stated (n = 9)

• staff questionnaires (n = 8)

It should be noted that a combination of methods of case ascertainment was used in some studies (e.g. patient self diagnosis and nurse diagnosis of SSI) however in these studies no comparison was made between the methods.

The CDC definition was the most commonly used definition of SSI and was applied in 38% (n = 28) of studies included. Other definitions used included authors' own (n = 8, 11% of studies) and other methods or methods unclear (n = 10, 14% of studies). No formal definition of surgical wound infection was provided in 26 reports (36%) of studies.

The duration of follow up within the post discharge surveillance programmes varied between 3 days post-discharge to several years; 30 days was the most common duration (n = 34, 50%). The use of a 30-day follow up point is consistent with the CDC definition of surgical wound infection (30 days is the required duration of follow up after operation if no implant is left in place or one year if implant is in place and the infection appears to be related to the operation) [6].

### Studies comparing the validity of alternative methods of post-discharge surgical site infection surveillance

Studies identified as validation studies were categorised by the methods used to assess validity and accuracy as previously described [1]. An overview of the characteristics of these studies is shown in Table 2.

**Table 2:** Characteristics of Included Studies Comparing Alternative Surveillance Systems

**Authors**	**Patient group**	**Surveillance method**	**Time point when surveillance undertaken**	**Response rate to surveillance**	**Infection rate detected**	**Notes**
Seaman & Lammers, 1991	433 patients with lacerations that were previously sutured, and had returned to the same county hospital for wound evaluation or suture removal.	i) Patient Interview by medical practitioner *Compared with Gold Standard * ii) Direct examination of wound by physician assistant or nurse.	Unclear. Patients had to return in 2–3 days for wound check and then at 5 to 14 days for suture removal	64.5% = 433 183 (27.2%) excluded due to incomplete forms 3 (0.4%) excluded because the definition of infection by the examiner was different to that defined for the study. 52 (7.7%) Patient had prior involvement in the study	4.8%	Many methodological details not reported in this study.
Sands et al 1996	5042 patients who underwent 5572 non obstetric procedures.	i) Computerised search of electronic records with codes indicative of SSIs, which were then confirmed by record review by ICPs. Compared with ii) Patient questionnaire and; iii) Surgeon questionnaire	i) Records were searched 30 days post-operatively. ii) 25^th^-32^nd ^postoperative day iii) 4–8 weeks post surgery.	i) 100% ii) 33.4% iii)79%	2%	Gold standard in this study comprised of wounds detected by any method and confirmed by case note review.
Sands et al, 1999	3636 patients undergoing 4086 non-obstetric surgical procedures.	i) Computerised search of electronic records with codes indicative of SSIs, which were then confirmed by record review by ICPs. Compared with ii) Model created from above data	i) 30 days post discharge ii) Unclear but uses above data.	i) 100% ii) Unclear	2.3%	Study was a substudy of Sands et al 1996, using a subgroup of the study population from and same gold standard as previous study.
Mitchell et al, 1999	1360 patients having undergone procedures chosen to represent the major elective procedures performed by cardiothoracic, vascular, abdominal, orthopaedic and gynaecological surgeons at the hospital. They included clean and 'contaminated' procedures for which the expected postoperative stay exceeded 5 days.	i) Patient questionnaire *Compared with * ii) Surgeon questionnaire	i) 28 days post-operatively. ii) At the time of postoperative review (exact time not stated)	i) 57.5% ii) 50.0%	6.91%	Gold standard unclear as some wounds that were detected by patients but not surgeons were reclassified as SSIs.
Whitby et al, 2002	343 patients chosen as a purposeful choice of high proportion of 'contaminated' and 'dirty' procedures.	i) Patient questionnaire *Compared with Gold standard * ii) Nurse diagnosis iii) Surgeon and Infection department physician/microbiologist assessed images of the wound	i) Questionnaire completed at 4 and 6 weeks ii) Weekly nurse visit with visual inspection of the wound, recording of symptoms from week one to six post-discharge iii) Not stated when the photos were taken that were assessed by these personnel.	i) 27 (7.8%) Inegible, unable, unwilling to participate. 225/316 (71.2%) completed questionnaire week 4. 190/316 (60.1%) completed questionnaire at week 6. ii) 290/316 (91.8%) followed up weekly iii) Not stated.	16.6%	High levels of disagreement between different health professionals were observed in this study suggesting that when one group can only make the assessment of wound infection based on photographic evidence this may not lead to accurate diagnoses.
Martini et al, 2000	1664 patients. The patient sample consisted of all surgical patients treated between May 1995 to December 1996.	i) Patient survey *Compared with Gold standard of * ii) Evaluation of hospital documents and treating consultants records	i) 3 months post-discharge ii) Not stated	i) 1535 (92%) ii) Not stated, however no mention of missing data.	1.1%	Unclear whether patients may have sought treatment for their infected wounds elsewhere and whether the timing of the survey to patients could have led to recall bias.
Sands et al, 2003	1352 Coronary Artery Bypass Grafts (CABG) procedures performed between June 1997 and March 2003	i) Administrative claims data and pharmacy dispensing records. Compared with ii) Examination of medical records for signs of infection using CDC criteria for wound infection by trained abstractor, further confirmed by physician epidemiologist, for a subset of patients that were at a higher probability of infection. N = 388 patients in this category	i) 180 days before surgery until 30 days post surgery. ii) Records for each of the 30 days post-surgery.	i) Not reported ii) 328 (85%) post-discharge information.	50.9%	Gold standard in this study comprised of wounds detected by any method and confirmed by case note review.

1) Comparison of different processes of case ascertainment of SSI, including case ascertainment by different health care professionals (no studies)

2) Assessment of patients' own ability to self diagnose wound infection, compared with health professional diagnosis (4 studies)

3) Studies of the validity of systems and examination of the feasibility of using routinely available data (e.g. antibiotic prescribing) (2 studies).

4) Validation reports of data capture methods, in particular, manual versus automated data entry (no studies)

#### 1. Validation of case ascertainment

We found none of these studies.

#### 2. Validation of patient self-diagnosis

Four studies validated patient self-diagnosis of surgical site infections after hospital discharge. [9-12] Two of these studies [9,10] were included in a previous review [1] and data were re-extracted for this review for completeness. These studies compared patient self-diagnosis with health professional diagnosis.

Seaman & Lammers [9] found that patients were unable to recognise infections in their own wounds. Of the 21 wound infections that were identified by health care professional assessment only 11 were detected by patients themselves, (a false negative diagnostic rate of 48%). However, the paper did not detail the questions asked of the patients.

In another study [10], both patients and surgeons agreed that wound infection was absent in 565 cases. Surgeons classified infection as present in 59 wounds whilst patients classified infection as present in 74 wounds; the researchers on further investigation then re-classified 23 wounds regarded as not infected by surgeons as infected – rejecting the gold standard in favour of patient assessment. Reasons for misclassification were given as surgical wound assessment preceding the development of infection and patients having reported their infection to someone other than their surgeon. False negative rates for patient assessment were also very low. Overall the agreement between surgeon and patient assessment was substantial, with a Kappa of 0.73. However, there was high proportion of missing data. The results of this study are difficult to interpret since surgeon and patient assessment did not coincide in time (and so were not assessing a wound in an identical 'state') and response rates were low. The results do provide some evidence that patients *may *be able to self diagnose wound infection with a reasonable level of agreement with the surgeon.

Whitby *et al *[11] analysed the validity of self-diagnosis post-discharge in Australia, by comparing self diagnosis by questionnaire with both Infection Control Nurse diagnosis and independent medical assessment of wound photographs for evidence of discharge and/or swelling. This study found that patients were unable to effectively self assess surgical site infections post discharge (positive predictive value of 29% – in other words only 29% of people who assessed themselves as having an infection actually had one). However, the negative predictive value for patient assessment was high (98% of patients who assessed themselves as not having an infection did not have one). Data presented in the publication did not allow for the calculation of the sensitivity and specificity of any of the methods compared with the gold standard; however sensitivity and specificity are required to make an assessment of the validity of the methods. Furthermore, both the positive and negative predictive values reported in this study are highly dependant upon the prevalence of infection in the population and as such cannot be generalised to other populations.

The fourth study [12] compared patient assessment with health care (clinic and general practitioner) data. Of the 92% of patients who responded, 64 (4.2%) reported having had an infection or inflammation of the wound. Scrutiny of clinic and general practitioner data identified 9 (0.5%) cases. It is not clear whether this disagreement is due to time lag bias or use of different criteria for diagnosis. No *a priori *definition of wound infection was described. It is unclear if all practitioners were using the same criteria by which a judgement of wound infection could be objectively and consistently made.

The findings of these 4 studies of patient self diagnosis were inconsistent; both high false positive and high false negative rates were reported.

#### 3. Studies of the validity of systems and examination of feasibility of using routinely available data

Three studies compared the validity of using existing data systems to identify patients with SSIs with prospective hospital based surveillance using slightly different comparators (variously questionnaires to patients and surgeons; a computerised algorithm and routine hospital surveillance [13-15].

The first study compared automated record screening plus physician review of records with both patient and surgeon questionnaires to detect SSIs [13]. This study included 5042 patients (all members of a health management organisation) who had undergone 5572 procedures and had electronic medical records from a single hospital in the US. The gold standard assessment comprised screening of routine medical data for diagnostic or treatment codes suggestive of SSI that occurred 30 days post-operatively. Subsequently full text records with the appropriate codes were assessed by two infection control physicians who classified the wound as infected or not according to the 1992 CDC definition. Any disagreements were resolved by a third surgeon assessor. This gold standard was compared with the results of patient questionnaires mailed between the 25^th ^and 32^nd ^postoperative day and surgeon questionnaires containing information on all surgical cases from the previous 4–8 weeks. If any additional SSIs were reported by either patients or surgeons, charts were reviewed again and checked by the infection control personnel. Patients returned only 33% of the questionnaires whilst surgeons returned 79% of questionnaires. Sensitivity of a positive patient response was 28% (in other words only 28% of patients who were diagnosed with a wound infection were able to detect it themselves) and the positive predictive value was 36% (in other words 36% of patients that self diagnosed as having a wound infection actually did have the condition). The sensitivity and positive predictive values of a positive surgeon responses were 24% and 19% respectively.

The authors of the first study then undertook further work using a subset of patients from the first study [13], including 3636 patients who had undergone 4086 procedures and had no SSI detected in the previous study prior to hospital discharge [14]. The authors used mathematical modelling to identify which insurance claims, diagnostics and treatment codes from routine data best identified patients with SSIs after discharge. They then compared these codes singly and in combination with the patient and surgeon questionnaire responses from their previous report.[13] The authors were able to detect 74% of SSIs in a subset of high risk procedures using only hospital discharge diagnosis codes plus pharmacy dispensing data with a specificity of 94%. Accepting a specificity of 92% improved the sensitivity from 74% to 92%.

A second study compared the validity of using existing data systems to capture patients with SSIs with prospective hospital based surveillance using NNIS criteria [15]. This study restricted the analysis to patients who were members of a specific health insurance plan with an infection probability of 0.1, resulting in 388 eligible patients from a total of 1352 Coronary Artery Bypass Graft patients in the United States.

Surveillance based on health insurance data identified approximately 50% more infections than did hospital-based surveillance and more than twice the number of infections that occurred post discharge. Sufficient detail was not available to calculate the specificity of either of the methods in this study.

There were methodological problems inherent in both of the studies; not least the lack of gold standard comparison and therefore no sense of the extent of misclassification by either system, neither of which involved purposeful patient examination post-discharge. Rather, the authors have taken the combined figure of positive results of infection irrespective of method of diagnosis to be the true positive rate. Whilst this may have increased the reported sensitivity of the tests, the ratio of difference between the two tests would remain the same.

In summary, two studies (3 reports) suggested that health insurance administrative data may detect more patients with SSIs than hospital based surveillance in the United States.

#### 4. Validation of data capture methods

No studies were found that compared a "gold standard" reference method with an existing data collection system only although there was some component of this in one of the studies found and described above [15].

All studies were then assessed for validity using the QUADAS tool. The result of this assessment is shown below in Table 3.

**Table 3:** Assessment of comparative studies using the QUADAS tool

**QUADAS Item**	**Sands (1996)**	**Sands (1999)**	**Sands (2003)**	**Whitby (2002)**	**Seaman & Lammers (1991)**	**Mitchell (1999)**	**Martini (2000)**
Was the spectrum of patients representative of the patient who will receive the test in practice?	Yes	Yes	No. CABG patients with a probability score of 0.1 of developing an SSI.	No. Chose moderate to high-risk patients only.	No. Only included patients with lacerations treated at A & E.	Yes	Unclear. Not enough information presented
Were the selection criteria clearly described?	Yes	Yes	Yes	No. Patients included if had a surgical wound that could be easily observed and if procedure undertaken had a moderate to high risk infection probability but not stated how this was calculated.	Yes	No.	No
Is the reference standard likely to correctly classify the target condition.	Unclear	Unclear	No. Relies on reporting, no observation.	Unclear. Different health professionals engaged in study had different levels of agreement.	Yes	Unclear	No.
Is the time period between the reference standard and index test short enough to be reasonably sure that the target condition did not change between the two tests?				Unclear. As authors did not specify what type of surgery was undertaken it is not known whether the follow-up period was sufficient to detect infections.	Unclear	No	Unclear
Did the whole sample or a random selection of the sample, receive verification using a reference standard of diagnosis?	Yes	Yes	Yes	Yes	Yes	No. Only those whose surgeon completed the questionnaire	Yes
Did patients receive the same reference standard regardless of the test result?	Yes	Yes	Yes	Yes	Yes	Yes	Yes
Was the reference standard independent of the index test (i.e. the index test did not form part of the reference standard)?	Unclear	Yes	No	Yes	Yes	Yes	Yes
Was the execution of the index test described in sufficient detail to permit replication of the test?	Unclear	Unclear	Yes	Yes	Yes	Unclear	Yes
Was the execution of the reference test described in sufficient detail to permit replication of the test?	Yes	Yes	No	Yes	Yes	Yes	Yes
Were the index test results interpreted without knowledge of the results of the reference test?	Unclear	Unclear	Unclear	Yes	Yes	Unclear	Yes
Were the results of the reference standard interpreted without knowledge of the results of the index test?	Unclear	Unclear	Unclear	Yes	No	Unclear	Yes
Were the same clinical data available when test results were interpreted as would be available when the test is used in practice?	No. The information available in this standard would not be available in the UK.	No. The information available in this standard would not be available in the UK.	No. The information available in this standard would not be available in the UK.	Yes	Yes	Yes	Yes
Were uninterpretable/Intermediate test results reported?	Unclear	Unclear	Unclear	Unclear	Unclear	Unclear	Unclear
Were withdrawals from the study explained?	Unclear	Unclear	Unclear	Unclear	Yes	Unclear	Unclear

Overall, there were some methodological limitations in all 6 comparative studies (7 reports) and the quality of reporting was also variable, as evidenced by the frequency of "unclear" responses to the QUADAS questions. The most common methodological limitation encountered in the studies was the lack of description of the time points at which assessments were made and a lack of description of the criteria used to define whether a wound infection was present.

### National audit

In total, 361 Infection Control personnel from 317 trusts or health boards were sent an audit form in May 2004, and asked to return the form irrespective of whether they were performing post discharge surveillance. Overall, 46% (n = 146) of trusts and health boards returned the audit form (only one response was counted in the numerator where multiple responses were received from single institutions within trusts or health boards). Of those trusts that responded, 29% (n = 42/146) reported performing some form of post-discharge surveillance and 71% (n = 104) said they were not.

Of the minority of trusts performing PDS, 14% (n = 6/42) reported doing so in all surgical areas. Otherwise, orthopaedics (33%, n = 14) was the speciality most commonly undertaking PDS for SSI with obstetrics (post-caesarean section) also being common (23%, n = 10). Other surgical procedures that were followed in smaller numbers were vasectomies, craniotomies, CABG, large bowel surgery, breast surgery, general surgery, hernia repair, vascular surgery and day surgery.

The most common methods of PDS reported in the audit were of routine clinical follow up (45% or n = 19/42) and direct observation of the wound (41% or n = 17/42) of positively responding trusts. An even greater number of respondents (50% or n = 21/42) reported using another method or a combination of methods. These included: giving forms to patients or primary care providers to return in 30 days; looking at hospital readmission data at 30 days post discharge; writing letters to, or telephoning General Practitioners to enquire as to any signs of infection post discharge or a combination of these and other methods. Other methods such as surgeon surveys, patient telephone or postal questionnaires were less frequently undertaken.

## Discussion

Fundamental to the conduct of surveillance is the need for a feasible, valid, reliable and standardised means of defining and ascertaining cases of surgical site infection, including those that occur post discharge. This review has shown that existing research evidence has not yet identified a feasible and robust means of ascertaining cases of SSI post discharge, principally because little research has been undertaken, and it has been frequently methodologically inadequate.

The optimum study design for evaluating the accuracy of diagnostic tests (post-discharge detection of SSI can be regarded as such a test) has been proposed to contain three key features:

1. a series of patients which represents an appropriate clinical spectrum;

2. patients receive both the new test and the reference or 'gold standard' test irrespective of the results of either test;

3. the reference or 'gold standard' should be measured independently of the new test.[16]

Studies of this kind allow for the calculation of test accuracy values of sensitivity and specificity, as well as feasibility, and it is this kind of study that is required to establish a method of accurately capturing data about post discharge SSIs.

Only six studies (seven reports) comparing alternative methods of surveillance for SSI post discharge were located. These studies compared:

• ascertainment of SSI by the capture of data from existing systems (claims and pharmacy dispensing data, medical records) with prospective hospital based surveillance using NNIS criteria [15]. This study found that data from existing administrative systems (in the USA) were better able to detect surgical site infections than routinely collected data.

• ascertainment of SSI by the capture of data from existing systems (claims and pharmacy dispensing data, medical records) with patient and surgeon questionnaires. This study found that data from existing administrative systems (in the USA) were better able to detect surgical site infections than either patients or surgeons [13,14]

• Surgeon questionnaire-based assessment of patient wound status compared with patient self-assessment of wound status by postal questionnaire [10]. This study found that there was substantial agreement between surgeons and patients regarding the status of their wounds – however, surgeon assessment data were missing for 50% of patients.

• Patient self-diagnosis by interview compared with health professional diagnosis found that patients were unable to diagnose infection or recognise signs of inflammation producing false negative rates of 48% [9].

• Infection control nurse diagnosis compared with patient self-assessment, surgeon diagnosis, infection department physician/microbiologist and patient recall of general practitioner antibiotic prescription [11]. This study found that patients were not able to adequately identify infected wounds (i.e., a high false positive rate). Further results in this study are uninterpretable since they were analysed as correlation rather than agreement, although it is noteworthy that correlation between methods was poor.

• Patient reported symptoms of infection with outpatient clinic physician diagnosis [12]. This study found differing rates of infection were detected via patient report and outpatient clinic follow up.

Two main issues arose from the comparative studies. Firstly, variations in data collection procedures and classification systems between countries limits comparability and prevents synthesis of the post discharge surveillance data. Many studies did not provide clear description of the criteria by which a diagnosis of infection was made and such information is crucial to the interpretation of the data. Secondly studies were undertaken in different patient populations (from low risk to very high risk) and the performance of a test will be influenced by the prevalence of infection in the population. Together these factors limit the usefulness and applicability of the evidence to the United Kingdom where vastly different data collection systems are in place.

The nature of potential surveillance methods depends on their purpose. If the purpose of the surveillance is to detect and treat SSIs in a timely manner after discharge then the system requires a mechanism for rapid alerting of healthcare professionals, probably instigated by the patient, based on a standard diagnostic criteria (such as the CDC definition). However, if the primary purpose is performance/outcomes monitoring then timeliness is less important than accuracy and thoroughness of data capture. One of the most promising methods of surveillance identified by this review was that of using automated screening of electronic records. However the applicability of this method beyond the USA and countries with similarly sophisticated data capture systems is unclear. In the UK for example, an integrated information system is currently being implemented across the National Health Service however, this implementation will take several years. If successful, post discharge surveillance of SSIs using clusters of diagnostic and treatment codes may be feasible. Such a system would require linkage of hospital and general practice data.

The accuracy of patient self diagnosis was variable across the studies and whilst current evidence does not yet support it as a valid method of case ascertainment for surveillance, it may be that if patients are asked the right questions they would be able to diagnose and report SSIs with an acceptable level of accuracy. Future research into methods of case ascertainment post discharge might usefully evaluate data capture from patients post discharge such as that recently described (but not evaluated) by Wilson *et al*[18].

The audit of current UK PDS SSI practice showed great variation in the methods and source of data that were being used. Multiple methods were commonly used including routine clinic follow up plus data from primary care providers. Orthopaedics and obstetrics were the most frequently observed surgical specialities undertaking PDS SSI. Importantly, whilst there are national policies to capture SSIs (for example the Health Protection Agency's mandatory orthopaedic surgical site infection surveillance which began on April 1^st ^2004), these schemes do not require post-discharge surveillance and do not give guidance as to how this could be undertaken. The nature of the post-discharge surveillance programme undertaken should depend on its aims. Surveillance of higher risk patient groups (i.e., those associated with a high incidence of SSI) enables identification of changes in patterns of infection and allows natural experiments of new interventions aimed at reducing infection rates. Surveillance of lower risk groups is also important to detect rates of preventable infection (i.e., that which is more likely to be associated with poor performance). Recent commentators have argued that surveillance of infection in all surgical specialties is feasible and cost effective[18]. Further research is ongoing to examine risk factors for surgical wound infection as well as the use of risk stratification in PDS which will further add to the body of knowledge in this area [19].

Finally evaluations of the surveillance programmes themselves are required so that its risks and benefits, including cost effectiveness, can be calculated. It has been suggested that any reduction in infection rates that may accrue as a result of the surveillance may require several years to develop, so a surveillance scheme may be cost effective only after a number of feedback cycles [18].

## Conclusion

As length of hospital stay after surgery continues to decline, a greater proportion of surgical site infections will occur after discharge; this presents challenges to the accurate monitoring of surgical infection rates [14]. More research on methods to measure surgical site infection rates after hospital discharge is needed. Preliminary work, using consensus techniques, should determine an appropriate reference (gold standard) method of case ascertainment post discharge and any potential new method of ascertainment (such as patient report using postal questionnaire) can be compared with this using established methods for comparing diagnostic tests. Where comprehensive, integrated (crossing primary and secondary care), electronic health records systems exist, there is evidence that they may offer a feasible and accurate method of surveillance.

## Competing interests

The author(s) declare that they have no competing interests.

## Authors' contributions

ESP, JED and NC undertook the systematic review and audit of infection control personnel. ESP, JED, NC and PJM participated in the design of the study and the drafting of the manuscript. All authors read and approved the final version of the manuscript.

## Pre-publication history

The pre-publication history for this paper can be accessed here:


